# Effects of Foliar Organic Selenium Application During the Main Season on Ratoon Rice Yield, Grain Quality, and Selenium Accumulation

**DOI:** 10.3390/plants14172758

**Published:** 2025-09-03

**Authors:** Jinfu Hu, Dehao Feng, Ziran Tang, Caise Ya, Xueer Lin, Kai Zhang, Xiong Yao

**Affiliations:** 1 College of Agriculture, Yangtze University, Jingzhou 434025, China; hujinfu0001@163.com (J.H.); 17354486072@163.com (D.F.); 18174923393@163.com (C.Y.); 18589902806@163.com (X.L.); 2 Chongqing Academy of Agricultural Sciences, Chongqing 401329, China; 2021051301027@stu.cqnu.edu.cn; 3 Hubei Climate Center, Wuhan 430074, China; 4 Second Geological Brigade of Hubei Geological Bureau, Enshi 445000, China

**Keywords:** chelating selenium, EDTA-Se, selenium speciation, selenium biofortification, yield components, processing quality, nutritional quality

## Abstract

With the growing global demand for rice and the urgent need to enhance sustainable production, ratoon rice systems and selenium (Se) biofortification technologies have become important strategies. This study investigated the effects of the foliar application of ethylenediaminetetraacetic acid Se (EDTA-Se) during key growth stages of the main rice season on the yield, grain quality, and Se accumulation in ratoon rice. Two rice varieties—Fengliangyouxiang-1 (FLYX1) and Jinliangyouhuazhan (JLYHZ)—were selected for a two-year field experiment. A systematic analysis was performed on yield components, processing quality, appearance quality, nutritional quality, and Se speciation. The results showed that under an equivalent total amount of spraying EDTA-Se, the best effect on improving the yield, grain quality, and grain Se content of ratoon rice was observed at the heading stage and seven days after full heading. This treatment increased ratoon season yield by 6.45%, primarily due to enhanced grain filling rate (GF) and spikelets per panicle (SP). Processing quality was significantly improved; milled rice rate (MR) increased by 5.59–6.24% in FLYX1 and 3.38–3.52% in JLYHZ, while appearance quality also improved, with chalky grain rate (CGR) decreasing by 21.51–22.93% in FLYX1 and 14.50–14.53% in JLYHZ. These improvements were closely associated with elevated protein content and increased accumulation of selenomethionine (SM). Notably, FLYX1 exhibited higher efficiency in converting selenium to organic forms, whereas JLYHZ showed a greater accumulation of inorganic selenium, highlighting genotype-specific responses. This study confirmed that the foliar application of EDTA-Se during key growth phases of rice during the main season can synergistically optimize yield and quality in ratoon rice while achieving Se biofortification and providing a theoretical basis and technical support for improving the quality and efficiency of ratoon rice, as well as producing Se-enriched ratoon rice.

## 1. Introduction

Rice (*Oryza sativa* L.) is the main food crop for human survival, and it is an important food source for more than 50% of the world’s population. Its global planting area is 163.2 million hectares, and its annual average production is 740.9 million tons [1,2]. With the growth of the global population, the demand for rice is also rising. Studies indicate that global rice production needs to increase by 116 million tons by 2035 to balance the supply and demand of rice [3]. In the context of global population growth and unbalanced rice supply, attention must be paid to the sustainable production of rice. One of the most highly regarded strategies for this is to increase the multiple season crop index, which can make more efficient use of existing land and increase the frequency of rice harvest through the ratoon rice planting system, thereby increasing the total yield of rice and contributing to the global rice supply [4,5].

Ratoon rice, as a special rice cropping system, refers to the method of harvesting one season of rice by using the surviving axillary buds on the rice pile after the harvest of the main season rice to germinate into panicles [6]. The promotion of ratoon rice planting not only effectively improves the multiple cropping index of paddy fields and increases the rice yield per unit area but also alleviates the contradiction between rice supply and demand, promoting the sustainable development of agriculture.

As an important trace element, selenium (Se) plays an indispensable role in human health. Se is the active center of many enzymes and participates in the body’s antioxidant defense system, which has a significant effect on improving human immunity and preventing diseases such as cancer [7,8]. By increasing the intake of Se-enriched agricultural products, diseases caused by Se deficiency can be prevented, and the intake of organic Se is considered to be safe and effective for humans [9,10]. Therefore, in the field of agricultural scientific research, the development of Se-enriched agricultural products has become one of the hot spots in current agricultural research; additionally, research on ratooning rice and Se has attracted increasing attention.

Through research, it has been found that rice has an excellent ability to be enriched with Se [11,12]. When Se fertilizer is applied to soil or leaves, the Se content of rice will increase significantly [13,14,15,16,17]. In a comparative study of two fertilization methods—soil application and foliar spraying—the results showed that spraying Se fertilizer directly onto rice leaves could more effectively increase the Se content of rice [13,18,19].

The process of Se absorption and accumulation in plants is closely related to the chemical form of Se fertilizer and the characteristics of the rice itself. The chemical structure of Se fertilizer is diverse, including selenate, selenite, organic Se, and nano-Se [15]. A large number of studies have explored the effects of Se, selenate, or selenite on Se uptake and accumulation in rice [20,21,22]. At the same time, studies have also revealed that there are genotypic differences in the absorption, accumulation, and distribution of Se in different rice varieties [12,23,24,25]. However, there are relatively few studies on the accumulation of Se in rice after the application of organic Se fertilizer. It is particularly noteworthy that organic Se, i.e., the form of Se that accumulates in rice, has a higher bioavailability and nutritional value, which is of great significance for improving rice quality and market competitiveness. At present, comparisons of the Se-enriched characteristics of different rice varieties mainly focus on Se-enriched rice and non-Se-enriched rice, while comparisons between the same type of rice (such as different indica rice varieties) are relatively rare [21,25]. However, although the importance of rice and Se has been widely recognized, existing studies on the application of Se in rice production have primarily focused on single-season or main-season crops, and the research on Se and ratoon rice is still in its infancy. Previous studies have shown that foliar application of Se in the main season can enhance the Se content and quality of rice in the main season [13,26]. Research on the quality of ratoon rice mainly focuses on yield, taste, and nutritional components, while the research on the accumulation of trace elements, especially Se, in ratoon rice and their effect on quality is still insufficient. This limits our in-depth understanding of the relationship between Se and ratoon rice, as well as restricting the further development of the Se-enriched ratoon rice industry. In light of this, it is of far-reaching significance to explore the relationship between Se and ratoon rice, as well as to study the effect of Se on the quality and grain Se content of ratoon rice, so as to enhance the added value of ratoon rice and promote the development of the Se-enriched ratoon rice industry.

The purpose of this study is to clarify the effects of different Se application periods on the yield formation and Se accumulation of ratoon rice, as well as to make up for the deficiency of the current application research of organic Se in ratooning rice systems. Although the effect of the foliar application of Se fertilizer on Se enrichment in rice has been confirmed, there is still a lack of systematic research on the dynamic effect of organic Se in ratoon rice production and its effect on the comprehensive quality of rice. To this end, a two-year field experiment was designed in this study. Two indica rice varieties were selected, and ethylenediaminetetraacetic acid-Se (EDTA-Se) was used as an exogenous Se source. In agriculture, EDTA not only prevents Se from being fixed or oxidized by soil but also optimizes the soil solution by chelating trace elements, thus significantly improving the bioavailability of elements and plant absorption efficiency and enhancing the comprehensive utilization rate of trace elements [27,28,29]. Foliar spraying was carried out at different growth stages of rice grown during the main season. The synergistic effect of organic Se on the yield improvement and quality optimization of ratooning rice was clarified by systematically analyzing the effects of Se application on yield composition, processing quality, appearance quality, nutritional quality, and the grain Se enrichment of ratoon rice. The results can provide theoretical support for the further promotion of the application of organic Se in rice production, the breeding of Se-efficient ratoon rice varieties, the optimization of precise Se application technology, and the development of functional Se-enriched rice, thus promoting the sustainable development of the Se-enriched ratoon rice industry.

## 2. Materials and Methods

### 2.1. Experimental Site and Materials

**Figure 1:**
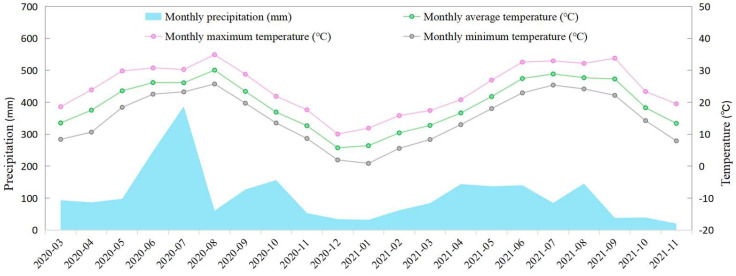
The temperature and precipitation during the experiment period from March 2020 to November 2021.

The experiment was conducted from March 2020 to November 2021 at the Yangtze University Teaching and Research Farm (30°32′–30°33′ N, 112°04′–112°05′ E) in Jingzhou City, Hubei Province. This region experiences a subtropical monsoon climate characterized by a mean annual precipitation of 1095 mm, an average temperature of 16.5 °C, and 1718 h of annual sunshine (20-year average: 2000–2020). The climatic conditions during the experimental period are detailed in Figure 1. The soil in the experimental field was calcareous fluvo-aquic soil. The soil samples were collected from the topsoil (20 cm) before the soil properties were analyzed [30]. The basic physical and chemical properties index is shown in Table 1. Fengliangyouxiang-1 (FLYX1) (provided by Hefei Fengle Seed Industry Co., Ltd., Hefei, China) and Jinliangyouhuazhan (JLYHZ) (provided by Hunan Jinjian Seed Industry Technology Co., Ltd., Changsha, China) were used as experimental materials.

### 2.2. Experimental Treatments

**Figure 2:**
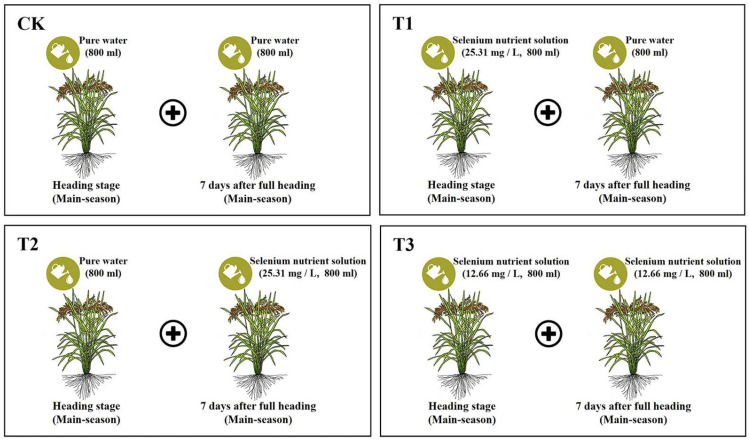
Schematic diagram of the experimental treatments for assessing the comprehensive effects of Se nutrient solution spraying on ratoon rice. Both 800 mL of Se nutrient solution and 800 mL of pure water were sprayed evenly onto the canopy leaves of rice in plots of equal area (plot dimensions: 4 m × 4.5 m = 18 m^2^).

A two-year field experiment was conducted using a randomized block design. EDTA-Se was used as a form of exogenous Se, and four treatments were set up by spraying canopy leaves, namely T1 (sprayed with 800 mL of Se nutrient solution (25.31 mg/L) during the heading stage and 800 mL of water 7 days after full heading), T2 (sprayed with 800 mL of water during the heading stage and 800 mL of Se nutrient solution (25.31 mg/L) 7 days after full heading), T3 (sprayed with 800 mL of Se nutrient solution at half concentration (12.66 mg/L) at both the heading stage and 7 days after full heading, respectively), and CK (sprayed with 800 mL of pure water sprayed at the heading stage and at 7 days after full heading, respectively), as shown in Figure 2. Three plots were set for each treatment, i.e., three replicates. The area of each plot was equal, at 4 m × 4.5 m = 18 m^2^. There were protective rows around the test plot to avoid boundary effects. Each treatment was managed uniformly, and different treatment concentrations were set at the heading stage and at 7 days after full heading, separated by partitions and sprayed with Se fertilizer, respectively.

### 2.3. Determination Indexes and Methods

#### 2.3.1. Agronomic Character

Before crop harvest, three 1 m^2^ sample points were collected in each plot, and the harvested rice was air-dried to the same moisture content (13.5%); then, the grain and biological yield were measured. At the same time, thirty plants were selected as samples to determine the yield, effective panicle number (PN), spikelet per panicle (SP), 1000-grain weight (GW), and grain filling rate (GF) (full grain number/spikelet number × 100%).

#### 2.3.2. Grain Quality

##### Processing Quality

Grain samples (50 g) were taken from each rice material; the brown rice rate (BR) and the milled rice rate (MR) were determined using a test huller (JLGJ-45, Jining Hanye Machinery Equipment Co., Ltd., Jining, China) and a heat-dissipating milled rice machine (LTJM-2099, Jilin Dingli Machinery Equipment Co., Ltd., Changchun, China).

##### Appearance Quality

Chalk grain rate (CGR): One hundred heads of rice were randomly selected, and a rice chalkiness observation instrument (JSE-II, Shanghai Tuoxi Electronic Technology Co., Ltd., Shanghai, China) was used to observe the existence of chalkiness (back white, abdominal white, or heart white), as well as to carry out related calculations.

Chalkiness degree (CD): In total, 30 grains of chalky rice were randomly selected, and the ratio of chalky area-to-total grain area was observed using a rice chalkiness observation instrument (JSE-II, Shanghai Tuoxi Electronic Technology Co., Ltd., Shanghai, China); the average value was calculated.

##### Eating Quality and Nutritional Quality

Amylose content (AC1): A total of 2–3 g of milled rice powder filtered through a 0.25 mm sieve was accurately weighed and placed in a 100 mL volumetric flask. Then, 1.0 mL 95% ethanol was added to disperse the sample, and 9.0 mL 1.00 mol/L NaOH solution was added. After shaking, the sample was heated in a boiling water bath for 10 min before being cooled to room temperature and diluted with deionized water to the scale line. Next, 5 mL of sample solution was transferred to another volumetric flask, and about 50 mL of distilled water was added, along with 1. 0 mL of 1 mol/L acetic acid and 1.50 mL of iodine solution; vortex mixing was used to ensure constant volume, and the samples were left in the dark for 20 min. In the blank group, 5.0 mL of 0.09 mol/L NaOH solution was used to replace the sample solution, and the subsequent coloration steps were completed simultaneously. Finally, the absorbance values of the samples and the blank group were measured using a spectrophotometer at a wavelength of 620 nm; measurements were made in parallel and were performed thrice.

Protein content: The mature rice was shelled and further ground into powder. The 2.5 g milled rice grains were mixed with 20 mL pre-cooled buffer (20 mmol/L Tris-HCl, pH 7.5; 250 mmol/L sucrose; 10 mmol/L EGTA; 1 mmol/L PMSF; 1 mmol/L DTT; and 1% TritonX-100). After shaking well, the mixture was centrifuged at 15,000× *g* for 15 min at 4 °C. The supernatant was preserved and mixed with 1/4 volume of pre-cooled 50% TCA in an ice bath for 30 min. After centrifugation at 15,000× *g* at 4 °C for 15 min, the protein particles were washed three times with cold acetone and were then vacuum dried. Next, 0.1 g of protein particles was mixed with 2 mL urea buffer (7 mol/L urea; 0.1 mol/L Tris-HCl, pH 7.5). The contents of albumin (AC2), glutelin (GC1), globulin (GC2), and total protein (TPC) were determined using the BCA protein assay, with BSA as the standard [31]. The alcoholic protein content (APC) was determined using Coomassie brilliant blue G-250 staining [32].

#### 2.3.3. Tetravalent Se (Se IV), Hexavalent Se (Se VI), and Total Se Content (TS)

During the preparation of the samples, the mature grains of rice treated with different types of Se were dried in a fume hood at 50 °C. The bran was then separated from the grain, and the seeds were ground into powder. The TS in the milled grain samples was determined using the AAS-HFS method. Before determination, 0.2 g of sample was digested with 3 mL concentrated HNO_3_ and 1 mL 30% H_2_O_2_ at 180 °C for 1.5 h. The digested product was reconstituted into 10 mL with Milli-Q water; then, the TS was determined via automatic sampling using AAS (Z2300, Hitachi, Ltd., Hitachi, Japan) and HFS (HFS-3,Hitachi, Ltd., Hitachi, Japan) [33].

At the same time, the content of Se IV and Se VI in grain samples was determined using the AAS-HFS method. Then, 1–2 g of sample was digested with 3 mL 4 mol/L HCl. The digestion process was carried out in boiling water at 100 °C for 10 min before being centrifuged at 2500× *g* at 4 °C for 10 min to remove the residue. The supernatant was reconstituted with Milli-Q water to a volume of 10 mL, and the AAS-HFS method was used [33].

#### 2.3.4. Selenomethionine Content (SM)

Water-soluble (‘free’) Se amino acids were extracted from 2 g of ground dried rice with 5 mL 20 mmol/L Tris-HCl (pH 7.5) at each determination timepoint. In the dark, the cells were shaken at 180 rpm and incubated at 37 °C for 2 h. Then, they were centrifuged at 10,000× *g* for 30 min at 4 °C before the selenoamino acids in the supernatant were quantified using HPLC-ESI-FTMS. In order to analyze the types of selenoamino acids bound to proteins, the protein extracts from rice were hydrolyzed using 6 mol/L HCl in the presence of 1% phenol at 110 °C in a vacuum for 24 h [34] before the composition of selenoamino acids in the hydrolysates was analyzed using HPLC-ESI-FT-ICR MS.

HPLC-ESI-FT-ICR MS analysis was performed using the UltiMate 3000 Basic Automated LC System (Dionex, Amsterdam, The Netherlands) and apex Qe FTMS instrument (Bruker Daltonics, Bremen, Germany). The HPLC system comprised an Agilent ZORBAX SB-C18 HPLC column (5 μm, 80 Å, 150 × 2.1 mm i.d.). HFBA was used as an ion-pair reagent in the mobile phase. The gradient was formed with 0.05% HFBA in H_2_O (A) and 0.05% HFBA in methanol (B), and the column flow rate was 0.3 mL/min. The 20 μL sample was injected into the chromatographic column and separated via stepwise gradient linear separation. The separation started at 2% B for 10 min and then increased from 5% to 40% B within 10 min before separating at 40% B for 5 min. The LC system was controlled by HyStar 3.2 (Bruker Daltonics).

The mass spectrometer was controlled by Apex Control version 1.0 (Bruker Daltonics). The photomultiplier tube voltage was 650 V, the source temperature was 120 °C, and the desolvation temperature was 250 °C. The acquisition range of Fourier transform mass spectrometry (FTMS) data was m/z 280 to 2500, and the average of the four spectra (13.7 s/spectrum) was taken with the same gradient. DataAnalysis 3.4 (Bruker Daltonics) was used to create a list of peaks from the raw data. The FTMS determination of SM was performed by scanning. The spectrum was obtained after parameter optimization [33,35]. Then, the intensity of selenoamino acids and their fragments was combined for quantification.

### 2.4. Statistical Analysis

In this experiment, the data were entered and organized using Microsoft Excel 2019 v16.0 (Microsoft Corporation, Redmond, WA, USA). Data were analyzed using ANOVA with SPSS 21.0 software (IBM Corp., Armonk, NY, USA). The mean of each treatment group was compared using the least significant difference (LSD) test, and the difference was statistically significant at *p* < 0.05. Graphical representation was conducted via GraphPad Prism 10.1.2 (GraphPad Software, San Diego, CA, USA).

## 3. Results

### 3.1. Yield and Yield Components

**Figure 3:**
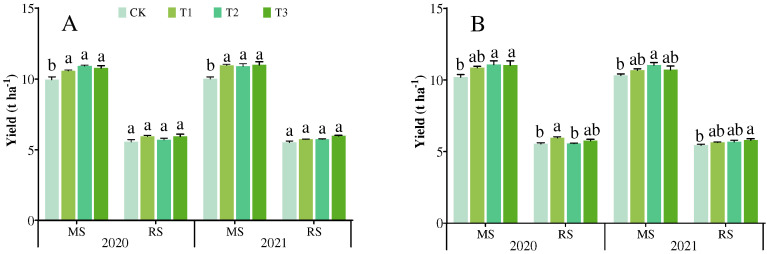
Effects of different treatments on the yield of different varieties of rice grown during the main season and the ratoon season. (**A**) shows the yield of Fengliangyouxiang-1 (FLYX1), and (**B**) shows the yield of Jinliangyouhuazhan (JLYHZ). MS: main season; RS: ratoon season. For each season, means followed by the same letters are not significantly different according to LSD.

The foliar application of Se fertilizer had different effects on the yield of rice grown during the main season and ratoon season, as well as having effects on the GF, GW, and SP of the ratoon season (Figure 3, Table 2 and Table 3). The results showed that different Se fertilizer spraying treatments were helpful in increasing rice yield, among which T3 treatment increased the yield of the ratoon season by 6.45% compared with CK treatment. Key yield components exhibited varied responses in each treatment. Specifically, GF increased across all treatments, with the best performance under T1 (9.07–9.61% in FLYX1; 7.20–7.58% in JLYHZ). SP was maximized in FLYX1 under T3 (7.61–7.80% increase), while GW improved only in JLYHZ (6.52–6.86% under T3). It is worth noting that the PN of the two rice varieties did not show significant differences over the two years.

### 3.2. Processing Quality and Appearance Quality

The BR, MR, CGR, and CD of rice grown during the ratoon season were different under various Se fertilizer spraying treatments (Table 4 and Table 5). The results showed that different Se fertilizer spraying treatments were helpful for improving the BR and MR of rice. Compared with CK, T1 treatment was the best in improving BR (6.37–6.39% in FLYX1; 2.34–2.67% in JLYHZ), while T3 treatment was the most effective in improving MR (5.59–6.24% in FLYX1; 3.38–3.52% in JLYHZ). In addition, chalkiness-related traits (CGR and CD) decreased most under T3, with FLYX1 showing a 21.51–22.93% reduction in CGR and 4.18–5.51% in CD, while JLYHZ exhibited a 14.50–14.53% lower CGR and 2.48–4.13% reduced CD compared to CK. These improvements demonstrate the role of Se in stabilizing grain structure and reducing internal defects.

### 3.3. Nutritional Quality and Eating Quality

The foliar application of Se fertilizer significantly increased the protein content of rice grain that was grown in the ratoon season (Table 6 and Table 7). Compared to CK, T3 maximized GC1 (9.53–10.48% in FLYX1; 5.71–6.00% in JLYHZ) and total protein content (TPC) (13.07–13.49% in FLYX1; 5.75–6.03% in JLYHZ). In addition, APC surged under T3 (64.18–64.43% in FLYX1) and T2 (53.91–55.45% in JLYHZ), indicating the enhanced solubility of functional proteins. There was no significant change in AC1, indicating that Se had little effect on grain starch composition.

### 3.4. Se Content in Grains

**Figure 4:**
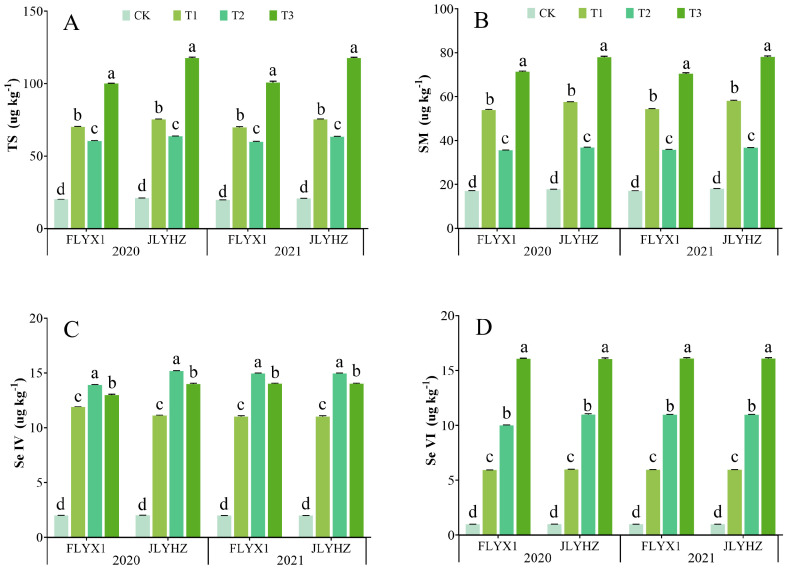
Total Se content (**A**), selenomethionine content (**B**), tetravalent Se content (**C**), and hexavalent Se content (**D**) in rice grains grown during the ratoon season under different treatments in 2020 and 2021. For different treatments of each variety, means followed by the same letters are not significantly different according to LSD.

The contents of SM, Se IV, Se VI, and TS in rice grown during the ratoon season were increased to different degrees under various Se fertilizer spraying treatments, and there were significant differences among varieties (Figure 4). For FLYX1, T3 treatment increased TS and SM by 397.26–410.41% and 311.23–316.84%, respectively, while the Se VI content increased by 1490.58–1511.29%. In comparison, JLYHZ had a higher increase in TS and SM under T3 treatment (457.02–463.85% and 331.52–336.27%), and Se VI accumulation (1503.31–1514.92%) exceeded that of FLYX1. In addition, T2 treatment significantly increased the Se IV content of JLYHZ (650.45–650.92%). These differences may be related to the differences in the expression of Se metabolism-related genes among varieties. In general, JLYHZ showed a higher inorganic Se accumulation potential, while FLYX1 was more efficient in organic Se transformation, suggesting that the application strategy of Se fertilizer should be optimized according to the characteristics of varieties in actual production to balance nutritional value and potential toxicity risk.

### 3.5. Correlation Analysis

**Figure 5:**
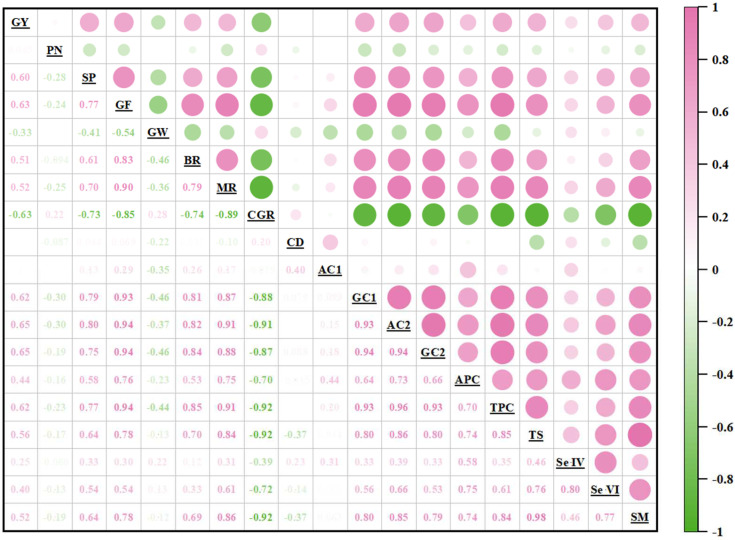
Correlation matrices between grain yield components and rice growth parameters. The related parameters include the yield of rice grown during the ratoon season (GY), panicle number (PN), spikelet per panicle (SP), grain filling rate (GF), 1000-grain weight (GW), brown rice rate (BR), milled rice rate (MR), chalk grain rate (CGR), chalkiness degree (CD), amylose content (AC1), glutenin content (GC1), albumin content (AC2), globulin content (GC2), alcoholic protein content (APC), total protein content (TPC), total Se content (TS), tetravalent Se content (Se IV), hexavalent Se content (Se VI), and selenomethionine content (SM), (n = 48). The size of the circle indicates the degree of correlation.

Correlation analysis was performed between the survey parameters, as shown in Figure 5. The results showed that the yield of rice grown during the ratoon season (GY) was positively correlated with TS, Se IV, Se VI, and SM, indicating that there was a synergistic effect between Se enrichment and yield improvement. CGR was significantly negatively correlated with protein content (GC1, AC2, GC2, APC, and TPC) and Se content (TS, Se IV, Se VI, and SM), indicating that the accumulation of Se and protein can effectively inhibit the formation of chalkiness and improve the appearance quality of grains. It is worth noting that protein content (GC1, AC2, GC2, APC, and TPC) is generally positively correlated with Se content (TS, Se IV, Se VI, and SM), further confirming the multi-dimensional improvement of Se on rice’s nutritional quality.

## 4. Discussion

### 4.1. Accumulation and Form of Se

In this study, by comparing and analyzing the Se content of rice grains in the regeneration season under different treatments, it was found that the application of EDTA-Se significantly increased the Se level in rice grains. This phenomenon may be attributed to the fact that part of the Se absorbed by rice in the main season is stored in roots and stubble tissues, and during the ratoon season, these Se reserves are reactivated and transported to newly developed tillers and grains through a phloem-mediated redistribution mechanism. Among them, T3 treatment (spraying EDTA-Se at both the heading stage and 7 days after full heading) had significant advantages in increasing the accumulation of TS and SM in grains. Se exists in plants in various chemical forms, primarily including selenite (Se IV), selenate (Se VI), SM, and selenocysteine (SeCys) [36]. These Se species differ in bioactivity and nutritional value, with organic forms such as SeMet exhibiting a higher bioavailability and safer assimilation for humans, while inorganic forms like Se IV and Se VI demonstrate a lower nutritional efficacy and potential toxicity [37,38]. Plants predominantly convert absorbed Se into organic species, particularly SeMet and SeCys, through biosynthetic pathways [17]. Studies have shown that under the condition of low Se content in soil, spraying an appropriate amount of inorganic Se on the leaves can significantly increase the TS of rice grains, as well as increase the proportion of organic Se [26,36,39,40]. However, excessive Se application inversely increases inorganic Se accumulation while reducing the levels of organic Se [41]. Previous studies have shown that crops supplied with sodium selenate mainly accumulate organic Se, while crops supplied with selenate mainly accumulate selenate [42]. Spraying Se at different growth stages of crops also affected Se accumulation in grains. Li et al. applied Se to millet during grain filling and jointing and found that millet treated with Se accumulated more Se in grains during grain filling [43]. Hao et al. found that the application of Se at the heading stage of oats led to an increase in Se accumulation in the grain part compared with the flowering stage [44]. In this study, we observed that under the condition of spraying the same amount of EDTA-Se on rice grown during the main season, the effect of spraying EDTA-Se at the initial heading stage and at 7 days after full heading was the most successful. Specifically, T3 significantly increased TS and SM, while the accumulation of inorganic Se (Se IV and Se VI) varied between varieties. JLYHZ showed a higher inorganic Se accumulation potential, while FLYX1 was more efficient in organic Se transformation. These results were similar to the study of Yuan et al. (2023) [36], who reported that split foliar applications of organic Se during critical growth stages maximize Se bioavailability by synchronizing Se supply with grain filling phases. This suggests that the two-stage spraying strategy of T3 optimizes the transport of Se from vegetative tissues to grains, which is conducive to synthesis of organic Se. During this period, moderate temperature and balanced precipitation increased the leaf absorption of Se by crops [45], which confirmed that the selection of a suitable growth period is very important for Se application in rice.

The efficient accumulation of organic Se may be related to its easy absorption and metabolic synergy. On the one hand, organic Se is more easily absorbed by roots and distributed to grains through transport systems [46]; on the other hand, it may have a synergistic effect with other nutrient elements in the metabolic processes of rice, which promotes the accumulation and stability of Se. In addition, the absorption and utilization ability of different types of rice for organic Se may also be different, which needs to be further explored in subsequent studies. The results of this study confirmed the effectiveness of organic Se as a Se source in promoting the absorption and accumulation of Se in rice grown during the ratoon season. With the increase in people’s understanding of the health function of Se, improving the Se content in food crops has become an important means of nutrition enhancement. Therefore, the results of this study provide strong support for further promoting the application of organic Se in rice production during the ratoon season.

### 4.2. Effects of Se Application on Yield and Yield Components

This study demonstrated that EDTA-Se application not only increased the yield of rice grown during the main season but also enhanced the yield of rice grown during the ratoon season, which was similar to the conclusions reported by Wu et al. (2024) [47]. Notably, the split application of EDTA-Se at the heading stage and at 7 days after full heading (T3) exhibited the most pronounced yield improvement (6.45% increase in the yield of rice grown during the ratoon season compared to that of CK). This could be attributed to the synchronization of Se supply with critical growth phases. In this study, the increase in yield was manifested not only through increased SP in individual panicles but more prominently through the overall improvement in GF. This effect was significantly positively correlated with the accumulation of organic SM (Figure 5), probably due to the promotion of Se-mediated carbohydrate transfer from stem to spike [47]. The climatic conditions during the experiment (Figure 1) also affected the results. The high temperature during the growth period of the rice grown in the main season accelerated the photosynthetic activity and redistribution of Se to the tillers of the rice grown during the ratoon season [48]. These findings suggest that organic Se application promotes critical physiological processes during rice growth, thereby elevating the comprehensive level of yield components. A number of studies have shown that Se can alleviate the damage to the cell membrane caused by oxidative stress via the activation of the antioxidant enzyme system in rice, enhance the synthesis of photosynthetic pigments and the efficiency of light energy conversion, and promote dry matter accumulation [49,50,51,52].

Additionally, the different performance observed between FLYX1 and JLYHZ in terms of GW and SP response (JLYHZ showed a significant GW increase under T3, while FLYX1 showed a significant SP increase) suggests that genetic factors may influence Se utilization efficiency, highlighting the necessity of variety-specific Se management strategies. The interaction between treatment and variety (Table 3) shows that organic Se enhances the sink strength of different genotypes through different pathways. The SP advantage of FLYX1 may reflect its stronger tillering ability under Se-mediated antioxidant activation, as proposed by Zhang et al. (2014) [50]. Notably, a correlation was identified between the effect of EDTA-Se application on grain Se content and yield improvement in rice grown during the ratoon season. As grain Se content increased, the yield of rice grown during the ratoon season exhibited a significant upward trend. This observation further supports the positive role of organic Se in promoting rice growth and yield enhancement. It also implies Se’s crucial function in rice’s physiological metabolism, which potentially involves multiple aspects. Wu et al. found that low concentrations of Se can enhance the respiration rate of mitochondria and the electron transport rate of chloroplasts, indicating that Se can also participate in the energy metabolism of rice [53]. Wu et al. demonstrated that Se exerts positive regulatory effects on rice growth and development for rice that was grown during the ratoon season [47]. Primarily, Se stimulates root system development in rice, enhancing root absorption capacity and enabling plants to more efficiently take up soil moisture and nutrients, thereby establishing a foundation for high yield [54,55,56]. Furthermore, Se enhances photosynthetic efficiency in ratoon rice, facilitating the conversion of light energy to chemical energy, thereby providing greater energy support for plant growth [47,57]. This may be due to the fact that Se absorbed through the foliage of rice grown during the main season is mainly stored in the stem base, root system, and residual vascular tissues, while Se from these sites provides a continuous supply for the growth and Se enrichment of rice grown during the ratoon season, either through direct transfer or soil release mechanisms. These combined effects significantly increase the panicle grain number, filled grain number, and seed setting rate, ultimately achieving yield enhancement.

### 4.3. Effects of Se Application on Grain Quality

In this study, the application of EDTA-Se had a positive effect on the quality characteristics of rice grown during the ratoon season. Specifically, Se application increased BR and MR, as well as reduced CGR and CD, of which T3 had the most significant effect (Table 4). These improvements were closely related to TPC (Figure 5). Se-enriched grains exhibited higher GC1 and AC2 levels (Table 6), which stabilize endosperm structure by filling intercellular voids, thereby reducing chalkiness [27]. For example, T3 reduced CGR by 21.51–22.93% in FLYX1 and 14.50–14.53% in JLYHZ, aligning with findings by Gao et al. (2024) [58], who attributed Se-induced quality enhancements to protein-mediated starch packing. The significant improvement of BR and Mr under T3 treatment may be due to the fact that sufficient rainfall during the ratoon season maintained soil moisture, promoted the transport of Se from stems to grains, and promoted the effect of Se on improving starch biosynthesis and reducing grain cracks during drying, resulting in a more esthetically pleasing and uniform grain shape, a brighter grain color, an improved transparency, and a reduced ventral whiteness [27,58]. These improvements in quality traits not only enhanced the processing and appearance quality of rice but also endowed it with a higher market value.

Furthermore, the varietal interaction further regulated quality outcomes: the MR of FLYX1 increased by 5.59–6.24% under T3, while the improvement of JLYHZ was smaller (3.38–3.52%). Despite JLYHZ having higher SM accumulation (Figure 4), its limited MR improvement may stem from its elevated inorganic Se proportion. Studies have shown that inorganic Se may disrupt protein/starch interactions, leading to looser endosperm structure [41], while the higher proportion of organic Se in FLYX1 likely strengthened the protein/starch matrix more effectively [26]. This highlights that varieties with high conversion efficiency of organic Se should be preferentially selected in quality-oriented breeding.

## 5. Conclusions

This study reveals the effects of spraying EDTA-Se at different stages of the growth of rice in the main season on a series of indicators such as yield, yield composition, grain quality, and grain Se accumulation of rice grown during the ratoon season. The results showed that under the condition of spraying the same amount of EDTA-Se on rice grown during the main season, spraying half of EDTA-Se at the heading stage and at 7 days after full heading had the best effect on improving the yield, yield composition, grain quality, and grain Se content of ratoon rice. In general, spraying EDTA-Se at the appropriate time on rice grown during the main season rice can more effectively improve the yield and quality of rice grown during the ratoon season. The application of EDTA-Se is economically feasible due to the increase in yield and the added value of Se-enriched rice, but more extensive promotion needs to be supplemented by detailed cost-effectiveness studies. The results of this study verified the effectiveness of organic Se as a Se source in promoting the absorption and accumulation of Se in rice and provided strong support for further promoting the application of organic Se in rice production. It is suggested that future studies can further compare the gene expression differences in enzymes and Se transporters in relation to Se metabolism at these growth stages to provide a new perspective on the mechanism and physiology of Se in plants.

## Figures and Tables

**Table 1:** Basic physical and chemical properties of the tested soil.

pH	Organic Matter (g/kg)	Total Nitrogen (g/kg)	Total Phosphorus (g/kg)	Total Potassium (g/kg)	Alkaline Nitrogen (mg/kg)	Available Phosphorus (mg/kg)	Available Potassium (mg/kg)	Total Selenium (mg/kg)	Available Selenium (mg/kg)
5.79	21.00	1.69	0.61	3.49	81.49	45.01	113.98	0.17	0.01

**Table 2:** Yield components of ratoon rice.

Year	Variety	Treatment	Panicle Number (PN)	Spikelet Per Panicle (SP)	Grain Filling Rate (GF)	1000 Grain Weight (GW)
					**(%)**	**(g)**
2020	FLYX1	CK	380.95 a	77.35 c	68.78 c	28.82 a
		T1	368.20 a	80.40 b	75.62 a	28.15 b
		T2	377.95 a	78.9 bc	71.11 b	28.40 b
		T3	374.33 a	83.39 a	75.56 a	28.23 b
		Mean	375.36	80.01	72.62	28.40
	JLYHZ	CK	373.60 ab	78.33 b	69.09 d	28.78 b
		T1	383.25 a	79.61 a	74.07 a	28.16 c
		T2	368.43 b	78.64 ab	71.98 c	28.64 b
		T3	365.09 b	79.49 a	73.10 b	30.61 a
		Mean	372.59	79.02	72.06	29.05
2021	FLYX1	CK	376.39 a	77.24 c	68.52 d	28.66 a
		T1	365.98 a	79.93 b	76.12 a	28.20 bc
		T2	378.30 a	79.55 b	70.83 c	28.44 ab
		T3	361.57 a	83.12 a	75.10 b	27.99 c
		Mean	370.56	79.96	72.64	28.32
	JLYHZ	CK	371.80 a	78.23 a	68.86 c	28.71 b
		T1	374.66 a	78.81 a	74.08 a	28.06 c
		T2	376.08 a	78.92 a	72.24 b	29.06 b
		T3	372.73 a	79.42 a	72.31 b	30.58 a
		Mean	373.81	78.84	71.87	29.10

Notes: FLYX1—Fengliangyouxiang-1 cultivar; JLYHZ—Jinliangyouhuazhan cultivar. For different treatments of each variety, the same lowercase letters indicate no significant difference, while different lowercase letters represent a significant difference at the level of *p* < 0.05 according to the least significant difference test (LSD 0.05).

**Table 3:** Analysis of variance (ANOVA) of panicle number (PN), spikelet per panicle (SP), grain filling rate (GF), and 1000-grain weight (GW).

ANOVA	Panicle Number (PN)	Spikelet Per Panicle (SP)	Grain Filling Rate (GF)	1000 Grain Weight (GW)
			(%)	(g)
Year (Y)	ns	ns	ns	ns
Variety (V)	ns	14.43 ***	28.26 ***	148.56 ***
Treatment (T)	ns	28.76 ***	466.17 ***	72.00 ***
Y × V	ns	ns	ns	ns
Y × T	ns	ns	ns	ns
V × T	ns	13.04 ***	55.90 ***	107.42 ***
Y × V × T	ns	ns	ns	ns

Notes: ***: *p* < 0.001; and ns: not significant.

**Table 4:** Processing quality and appearance quality of rice grown during the ratoon season.

Year	Variety	Treatment	Brown Rice Rate (BR)	Milled Rice Rate (MR)	Chalk Grain Rate (CGR)	Chalkiness Degree (CD)
			(%)	(%)	(%)	(%)
2020	FLYX1	CK	74.85 c	55.52 c	12.18 a	2.67 c
		T1	79.61 a	57.64 b	10.42 c	3.12 a
		T2	75.60 c	55.67 c	11.25 b	2.76 b
		T3	77.86 b	58.63 a	9.39 d	2.53 d
		Mean	76.98	56.87	10.81	2.77
	JLYHZ	CK	75.48 b	55.71 c	11.71 a	2.47 b
		T1	77.49 a	57.66 a	10.89 c	2.44 bc
		T2	76.15 ab	56.60 b	11.39 b	2.98 a
		T3	76.59 ab	57.67 a	10.01 d	2.41 c
		Mean	76.43	56.91	11.00	2.57
2021	FLYX1	CK	74.50 c	55.50 d	11.98 a	2.65 c
		T1	79.26 a	58.22 b	10.37 c	3.11 a
		T2	75.06 c	56.20 c	11.35 b	2.77 b
		T3	78.26 b	58.97 a	9.40 d	2.54 d
		Mean	76.77	57.22	10.78	2.77
	JLYHZ	CK	75.80 b	55.80 c	11.65 a	2.50 b
		T1	77.57 a	57.45 a	10.81 c	2.48 b
		T2	75.81 b	56.70 b	11.27 b	2.97 a
		T3	77.22 a	57.69 a	9.96 d	2.40 c
		Mean	76.60	56.91	10.93	2.59

Notes: FLYX1—Fengliangyouxiang-1 cultivar; JLYHZ—Jinliangyouhuazhan cultivar. For different treatments of each variety, the same lowercase letters indicate no significant difference, while different lowercase letters represent a significant difference at the level of *p* < 0.05 according to the least significant difference test (LSD 0.05).

**Table 5:** Analysis of variance (ANOVA) of brown rice rate (BR), milled rice rate (MR), chalk grain rate (CGR), and chalkiness degree (CD).

ANOVA	Brown Rice Rate (BR)	Milled Rice Rate (MR)	Chalk Grain Rate (CGR)	Chalkiness Degree (CD)
	(%)	(%)	(%)	(%)
Year (Y)	ns	4.46 *	ns	ns
Variety(V)	ns	ns	12.42 **	354.44 ***
Treatment (T)	73.56 ***	206.00 ***	388.05 ***	337.81 ***
Y × V	ns	4.41 *	ns	ns
Y × T	ns	ns	ns	ns
V × T	14.67 ***	21.77 ***	21.92 ***	309.98 ***
Y × V × T	ns	ns	ns	ns

Notes: *: *p* < 0.05; **: *p* < 0.01; ***: *p* < 0.001; ns: not significant.

**Table 6:** Nutritional quality of rice grown during the ratoon season.

Year	Variety	Treatment	Amylose Content (AC1)	Glutenin Content (GC1)	Albumin Content (AC2)	Globulin Content (GC2)	Alcoholic Protein Content (APC)	Total Protein Content (TPC)
			(%)	(%)	(%)	(%)	(%)	(%)
2020	FLYX1	CK	19.51 b	7.23 c	0.33 d	0.33 c	0.11 d	7.90 c
		T1	20.58 a	7.89 a	0.46 b	0.37 a	0.14 b	8.86 a
		T2	20.30 a	7.34 b	0.37 c	0.34 b	0.13 c	8.19 b
		T3	20.32 a	7.91 a	0.47 a	0.37 a	0.18 a	8.93 a
		Mean	20.18	7.59	0.41	0.35	0.14	8.47
	JLYHZ	CK	20.36 b	7.19 d	0.33 c	0.33 b	0.11 d	8.02 c
		T1	20.90 b	7.50 b	0.38 b	0.35 a	0.16 b	8.44 a
		T2	22.34 a	7.34 c	0.37 c	0.34 b	0.17 a	8.20 b
		T3	19.61 c	7.60 a	0.39 a	0.35 a	0.15 c	8.49 a
		Mean	20.80	7.41	0.37	0.34	0.15	8.29
2021	FLYX1	CK	19.67 b	7.17 c	0.33 d	0.33 c	0.11 d	7.89 d
		T1	20.66 a	7.93 a	0.46 b	0.37 a	0.14 b	8.89 b
		T2	20.62 a	7.35 b	0.37 c	0.34 b	0.13 c	8.12 c
		T3	20.48 a	7.95 a	0.47 a	0.37 a	0.18 a	8.95 a
		Mean	20.36	7.60	0.41	0.35	0.14	8.46
	JLYHZ	CK	20.56 b	7.16 c	0.33 c	0.33 b	0.11 d	7.97 c
		T1	21.01 b	7.59 a	0.38 b	0.35 a	0.16 b	8.42 a
		T2	21.97 a	7.32 b	0.37 c	0.34 b	0.17 a	8.24 b
		T3	19.89 c	7.60 a	0.39 a	0.35 a	0.15 c	8.45 a
		Mean	20.86	7.42	0.37	0.34	0.15	8.27

Notes: FLYX1—Fengliangyouxiang-1 cultivar; JLYHZ—Jinliangyouhuazhan cultivar. For different treatments of each variety, the same lowercase letters indicate no significant difference, while different lowercase letters represent a significant difference at the level of *p* < 0.05 according to the least significant difference test (LSD 0.05).

**Table 7:** Analysis of variance (ANOVA) of amylose content (AC1), glutenin content (GC1), albumin content (AC2), globulin content (GC2), alcoholic protein content (APC), and total protein content (TPC).

ANOVA	Amylose Content (AC1)	Glutenin Content (GC1)	Albumin Content (AC2)	Globulin Content (GC2)	Alcoholic Protein Content (APC)	Total Protein Content (TPC)
	(%)	(%)	(%)	(%)	(%)	(%)
Year (Y)	ns	ns	4.93 *	ns	ns	ns
Variety (V)	50.23 **	266.10 ***	2971.35 ***	409.61 ***	756.00 ***	206.79 ***
Treatment (T)	59.66 **	651.89 ***	4350.78 ***	843.65 ***	7778.00 ***	797.34 ***
Y × V	ns	ns	ns	ns	ns	ns
Y × T	ns	5.66 **	ns	ns	ns	ns
V × T	38.23 **	71.83 ***	1220.14 ***	139.06 ***	3213.49 ***	146.81 ***
Y × V × T	ns	ns	ns	ns	ns	ns

Notes: *: *p* < 0.05; **: *p* < 0.01; ***: *p* < 0.001; ns: not significant.

## Data Availability

The datasets used and/or analyzed during the current study are available from the corresponding author upon reasonable request. The data are not publicly available due to the need for data confidentiality for subsequent research.
